# A Trace Element–Ulcer Map: Decoding Micronutrient–Ulcer Relationships Through Genetic Architecture and Pleiotropy‐Aware Inference

**DOI:** 10.1002/fsn3.71094

**Published:** 2025-10-31

**Authors:** Xueyao Cai, Weidong Li, Wenjun Shi, Can Liu, Yuchen Cai, Jianda Zhou

**Affiliations:** ^1^ Department of Plastic Surgery The Third Xiangya Hospital of Central South University Changsha Hunan China; ^2^ Department of Plastic and Reconstructive Surgery, Shanghai Ninth People's Hospital Shanghai Jiao Tong University School of Medicine Shanghai China

**Keywords:** circulating micronutrients, gastric ulcer, Mendelian randomization, putative causality, zinc

## Abstract

The contribution of circulating micronutrients to ulcer susceptibility remains poorly defined across anatomical sites. In this study, we constructed a systematic trace element–ulcer map by integrating genetic‐instrumented inference, pleiotropy‐aware modeling, and heterogeneity‐sensitive clustering. Summary‐level data were obtained from large‐scale genome‐wide association studies (GWAS) encompassing 10 circulating micronutrients (calcium, iron, zinc, copper, magnesium, selenium, carotene, vitamin B12, vitamin C, and vitamin D) and nine ulcer phenotypes (corneal ulcer, recurrent oral aphthae, esophageal ulcer, gastric ulcer, duodenal ulcer, vaginal/vulvar ulcer, decubitus ulcer, lower limb ulcer, and chronic skin ulcer), covering more than 3 million individuals of European ancestry. Our two‐sample Mendelian randomization (MR) analysis identified genetically elevated zinc levels as a risk factor for gastric (OR: 1.141, 95% CI: 1.060–1.228, *p* = 4.57 × 10^−4^) and esophageal ulcers, but inversely associated with vaginal/vulvar ulcer risk. Protective effects were observed for iron with gastric ulcers and calcium with duodenal ulcers. Carotene and magnesium were nominally associated with increased risk of oral aphthae and vaginal ulcers, respectively. To refine these associations, we applied Causal Analysis Using Summary Effect estimates (CAUSE) and MR‐Clust. While CAUSE did not confirm robust putative causal relationships in most pairs, MR‐Clust uncovered three distinct SNP clusters in the vitamin C‐corneal ulcer pair, indicating potential mechanistic heterogeneity. These pleiotropy‐aware and cluster‐based approaches enhanced the interpretability of borderline signals and revealed genetic heterogeneity beyond mean‐effect estimates. Collectively, this study offers a panoramic view of trace element‐ulcer relationships and prioritizes zinc as a key candidate for further mechanistic exploration in gastric ulcer pathogenesis. Our integrative framework may serve as a foundation for future etiological and nutritional intervention studies.

## Graphical abstract

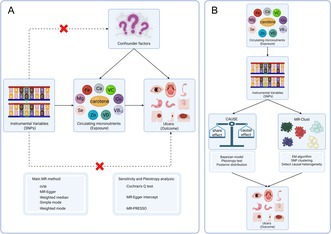

## 1 Introduction

Ulcers are chronic breaches of the skin or mucosal barrier that can occur in various anatomical regions, including the cornea, gastrointestinal tract, genital epithelium, and peripheral skin (Lanas and Chan 2017; Mauskar et al. 2020; Stechmiller 2010; Ung et al. 2020). Regardless of their location, ulcers can cause pain, infection, delayed healing, and considerable healthcare burden. While their etiologies are diverse, ranging from ischemia and infection to autoimmune conditions and mechanical pressure, micronutrient imbalance has emerged as a potentially modifiable factor contributing to ulcer development and progression.

Micronutrients, including trace elements (e.g., zinc, selenium, and iron) and vitamins (e.g., vitamin C, B12, and D), play critical roles in tissue regeneration, immune modulation, redox homeostasis, and epithelial integrity. Clinical studies have shown that supplementation with vitamin C, copper, selenium, and zinc can accelerate the healing of pressure ulcers, while vitamin D, vitamin E, and magnesium benefit diabetic foot ulcers (Afzali et al. 2019; Razzaghi et al. 2017; Van Anholt et al. 2010). However, such studies focus on wound repair rather than on primary ulcer prevention. Whether baseline circulating levels of these micronutrients increase or decrease ulcer risk remains poorly understood.

Historical insights from traditional Chinese medicine suggest that elemental imbalances may contribute to disease susceptibility. The saying “food surpasses medicine in nourishing the body” reflects an early recognition of the health consequences of dietary micronutrients. Ancient Daoist texts even propose that imbalanced grain consumption may trigger various ailments. Modern ecological research reinforces these ideas: for example, selenium‐deficient soil has been linked to Keshan disease in China (Tan et al. 2002), low zinc environments affect crop quality and human health in India (Shukla et al. 2021), and iodine‐deficient inland regions have increased thyroid disease prevalence (Delange 2002). These examples suggest that environmental micronutrient distribution may have measurable impacts on human disease, including ulcers.

Yet directly testing whether micronutrient levels cause ulcers is challenging. Randomized controlled trials (RCTs) that manipulate micronutrient status until ulcers develop would raise ethical concerns. Instead, Mendelian randomization (MR) for genetic‐instrumented inference offers an alternative approach. Genetic variants such as single‐nucleotide polymorphisms (SNPs), which influence steady‐state micronutrient concentrations, can be used as proxies to infer long‐term biological effects. Because genotypes are randomly assigned at conception, they are generally free from reverse causation and major confounding. This strategy enables the estimation of putative causal relationships between micronutrient exposure and ulcer outcomes. Moreover, recent methodological advances extend beyond traditional estimators. Models such as Causal Analysis Using Summary Effect estimates (CAUSE) explicitly account for pleiotropy—where genetic variants influence both the exposure and outcome through shared or independent pathways—offering pleiotropy‐aware inference (Morrison et al. 2020). Meanwhile, clustering‐based approaches such as MR‐Clust can identify distinct SNP groups, revealing potential mechanistic heterogeneity across genetic instruments (Foley et al. 2021).

Here, leveraging genome‐wide association summary statistics from up to 65,000 participants for 10 circulating micronutrients and over 400,000 individuals for nine ulcer phenotypes, we aimed to construct a comprehensive trace element–ulcer map through multilayered genetic inference. First, we systematically screened 90 micronutrient–ulcer pairs using genetically instrumented causal inference to identify potential associations. To evaluate the robustness of these findings, we applied CAUSE, a pleiotropy‐aware framework that accounts for both correlated and uncorrelated horizontal pleiotropy. Furthermore, to explore possible mechanistic heterogeneity, we used MR‐Clust to detect SNP clusters with distinct effect patterns. By integrating results across these complementary methods, we sought to distinguish putative causal, pleiotropic, or null relationships and to provide a structured view of how trace elements may contribute to the pathogenesis of diverse ulcerative conditions.

## 2 Methods

### 2.1 Research Design

**FIGURE 1:**
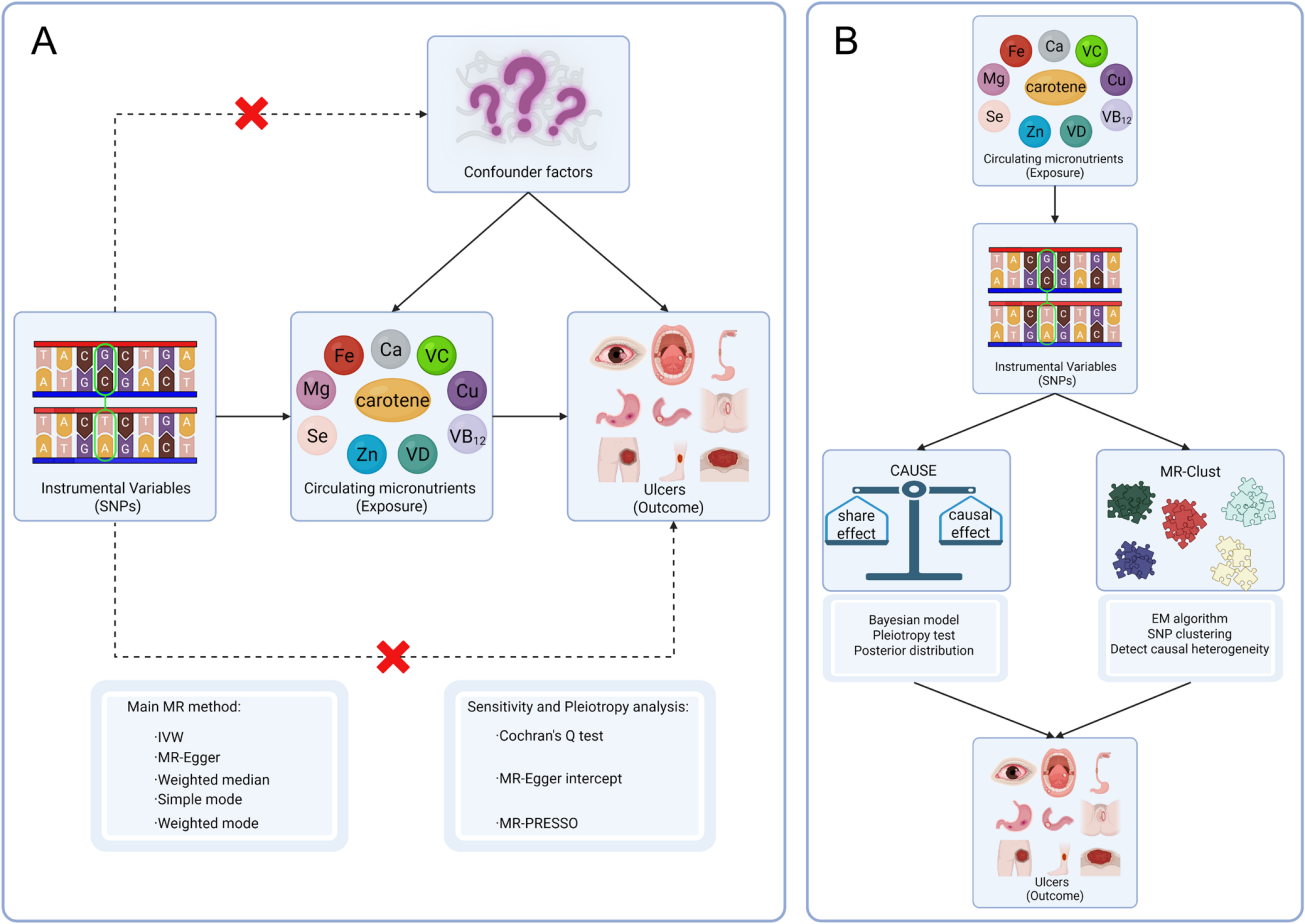
Study overview and Mendelian randomization (MR) model. (A) A two‐sample MR study was conducted using publicly available summary statistics from 19 genome‐wide association studies (GWAS): 10 circulating micronutrients for exposures and nine ulcer types for outcomes, followed by sensitivity analysis. (B) MR‐CAUSE and MR‐Clust were used, accounting for pleiotropy and heterogeneity. This figure was created in BioRender (Li, W. 2025, https://BioRender.com/f3dcnya).

A two‐sample MR study was conducted using publicly available summary statistics from 19 genome‐wide association studies (GWAS): 10 for exposures and nine for outcomes, followed by sensitivity analysis and MR‐CAUSE and MR‐Clust to account for pleiotropy and heterogeneity (Figure 1). Both exposure and outcome cohorts were limited to individuals of European ancestry to mitigate bias from population stratification. The study follows the following principles: IVs are directly associated with circulating micronutrient concentrations; IVs should remain unaffected by both known and unknown confounding factors; IVs exclusively influence ulcers via circulating micronutrient concentrations. All data used in this study are publicly accessible from the original studies with appropriate participant consent and ethical approval. Consequently, ethical approval from an institutional review board was deemed unnecessary for the current investigation.

### 2.2 Data Regarding the Genetically Predicted Levels of Circulating Micronutrients

We conducted a search for published GWAS using the IEU OpenGWAS database (https://gwas.mrcieu.ac.uk/datasets/). In this study, 10 micronutrients of potential interest were identified: calcium (dataset ukb‐b‐8951; *n* = 64,979), iron (dataset ukb‐b‐20,447; *n* = 64,979), zinc (dataset ieu‐a‐1079; *n* = 2603), copper (dataset ieu‐a‐1073; *n* = 2603), magnesium (dataset ukb‐b‐7372; *n* = 64,979), selenium (dataset ieu‐a‐1077; *n* = 2603), carotene (dataset ukb‐b‐16,202; *n* = 64,979), vitamin B12 (dataset ukb‐b‐19,524; *n* = 64,979), vitamin C (dataset ukb‐b‐19,390; *n* = 64,979), and vitamin D (dataset ukb‐b‐18,593; *n* = 64,979) (Table S1). For primary MR analysis, we incorporated independent SNPs with low linkage disequilibrium (LD) (*r* ^2^ < 0.001 within 10,000 kb windows) that were associated (*p* ≤ 1E−05) with blood levels of each micronutrient.

To mitigate the impact of confounding factors, we assessed the horizontal pleiotropy of each IV using the PhenoScanner database. F statistics are also used to assess weak IV bias. The F statistic is calculated using the formula: F = *R* ^2^ × (N‐2)/(1‐*R* ^2^), where N represents the sample size, and R^2^ represents the proportion of exposure variance explained by IVs. A calculated F statistic exceeding 10 indicates that the genetic variant used is a robust IV (Burgess et al. 2011; Burgess et al. 2016). In addition, we conducted leave‐one‐out analyses for micronutrients containing more than two SNPs. This was carried out to assess the robustness of inverse‐variance weighted (IVW) estimates and to identify any specific SNP that may be driving the association, which could potentially be attributed to pleiotropy (Davies et al. 2018).

### 2.3 Data Regarding the Genetically Predicted Levels of Ulcers

We utilized the publicly available summary statistics from FinnGen R10 (https://r10.risteys.finngen.fi/) for the following ulcers: corneal ulcer (ncase = 6118, ncontrol = 390,647), recurring oral aphthae (ncase = 3958, ncontrol = 408,223), esophageal ulcer (ncase = 2230, ncontrol = 350,064), stomach ulcer (ncase = 6459, ncontrol = 350,064), duodenal ulcer (ncase = 3795, ncontrol = 350,064), vaginal/vulvar ulcer (ncase = 618, ncontrol = 205,362), decubitus ulcer (ncase = 1621, ncontrol = 385,509), lower limb ulcer (ncase = 4101, ncontrol = 385,509), and chronic skin ulcer (ncase = 2001, ncontrol = 385,509). In addition, we performed Cochran's Q statistical test to assess heterogeneity between the two cohorts regarding the genetic instruments used for the outcomes.

### 2.4 Two‐Sample MR Analysis

Following the harmonization of SNPs in the dataset to ensure consistency of alleles, we conducted the two‐sample MR analysis. We calculated the Wald ratio for each SNP, defined as the SNP‐outcome association divided by the SNP‐exposure association. When multiple SNPs are available for a micronutrient, the fixed‐effect IVW method is employed as the primary approach to estimate the effect of exposure on outcomes (Burgess et al. 2013). This method is like the meta‐analysis of the effects of a single SNP on outcomes. It yields the most robust causal estimation and is relatively sensitive to pleiotropy. MR‐Egger, weighted median (WM), simple mode, and weighted mode were also used for MR analysis. All reported associations represent an odds ratio (OR) for outcome per standard deviation (SD) increase in genetically predicted circulating micronutrient concentrations. MR analyses were performed separately for GWAS results from the UK Biobank and FinnGen.

If there is heterogeneity in IVs, the results may be biased. Therefore, we conducted Cochrane's Q‐test to assess the level of heterogeneity, where *p* < 0.05 indicates a high level of heterogeneity (Greco et al. 2015). This test was conducted only when two or more variants were available. To evaluate horizontal pleiotropy effects, we used the *p* value of the MR‐Egger regression intercept (Bowden et al. 2015). The MR‐Egger method can detect potential pleiotropy and provides a more conservative estimate of causal effects by adjusting for pleiotropy. It allows for the possibility that certain SNPs may influence the outcome through a distinct pathway from exposure. A non‐zero intercept term suggests that not all included instruments are valid, potentially biasing the standard estimates (i.e., IVW). A *p* < 0.05 suggests a significant pleiotropy bias (Verbanck et al. 2018).

### 2.5 MR‐CAUSE Analysis

To further examine the robustness of observed causal associations and account for potential horizontal pleiotropy, we applied the MR‐CAUSE method (Morrison et al. 2020). CAUSE is a Bayesian framework that compares three models: the null model (no effect), the sharing model (shared pleiotropic effects without causality), and the causal model (causality with pleiotropy). The model comparison is based on the expected log pointwise predictive density (ELPD), and the causal effect is estimated through the posterior distribution of the gamma parameter, along with a 95% confidence interval (CI).

We applied CAUSE to five exposure–outcome pairs that demonstrated suggestive evidence of causality in two‐sample MR, including zinc–stomach ulcer, zinc–esophageal ulcer, magnesium–vaginal/vulvar ulcer, calcium–duodenal ulcer, and vitamin C–corneal ulcer. For each pair, SNP–exposure and SNP–outcome summary statistics were harmonized and filtered to remove palindromic SNPs and those with ambiguous alleles. We used the cause R package (v1.2.0) with default priors. LD pruning was performed using an *r* ^2^ threshold of 0.01 within a 10,000 kb window, as recommended. The prior on the proportion of variants with pleiotropic effects (q) was set to a uniform distribution on [0, 1]. We ran the cause function with default settings and computed the ELPD difference (ΔELPD) between the causal and sharing/null models. A positive ΔELPD suggests a better fit of the causal model. A detailed MR‐CAUSE R code is available on GitHub (https://github.com/kevinkevinkevin666/code) and Supporting Information.

### 2.6 MR‐Clust Analysis

To further explore the potential heterogeneity of instrument SNPs and to identify distinct clusters of variants that may represent different causal mechanisms, we performed MR‐Clust analysis. MR‐Clust is a model‐based clustering method that groups SNPs based on their joint distribution of genetic associations with both exposure and outcome. This approach allows for the identification of variant clusters consistent with different causal effects or confounding structures (Foley et al. 2021).

We applied MR‐Clust using the “MRClust” R package to all exposure–outcome pairs showing suggestive evidence of causality in two‐sample MR. SNPs were retained if they had nonzero effects on the exposure. For each analysis, SNPs were grouped into clusters, and the posterior cluster probabilities were estimated. Clusters with consistent directional effects and high posterior probability were interpreted as putative causal signals. Default parameters were used unless otherwise specified, including the expectation–maximization algorithm for parameter estimation and a tolerance of 1e‐5 for convergence. Results were visualized using effect size scatterplots with cluster assignments. A detailed MR‐Clust R code can be found on GitHub (https://github.com/kevinkevinkevin666/code) and Supporting Information.

## 3 Statistical Analysis

We calculate the statistical power of the MR analysis using the specialized network tool mRND (https://shiny.cnsgenomics.com/mRND/) (Brion et al. 2013). Power estimation for circulating micronutrient concentrations was based on a Type I error of 5%. Key parameters in the calculation included sample size, case proportion, outcome OR, and *R* ^2^. All data analysis in this study was conducted using R software (v4.3.1) (Hemani et al. 2018). The *p* values were corrected for multiple comparisons using the Bonferroni method, with a significance threshold set at *p* < 0.005 (0.05/10 exposures). The *p* value of 0.005 to 0.05 is considered nominally significant. Visualizations for forest plots, scatter plots, funnel plots, and leave‐one‐out sensitivity plots were generated using R version 4.3.1 with the ggplot2 package.

## 4 Results

### 4.1 Causal Effects of Circulating Micronutrient Concentrations on Ulcers

In total, this study identified 224 SNPs associated with 10 circulating micronutrients as IVs. Table S1 presents the information on SNPs associated with circulating micronutrient concentrations. The F statistics of all SNPs are greater than 10, indicating the absence of weak IV bias in our MR analysis. Detailed information on the correlation of IVs with 10 circulating micronutrient concentrations and ulcers can be found in Table S2.

**FIGURE 2:**
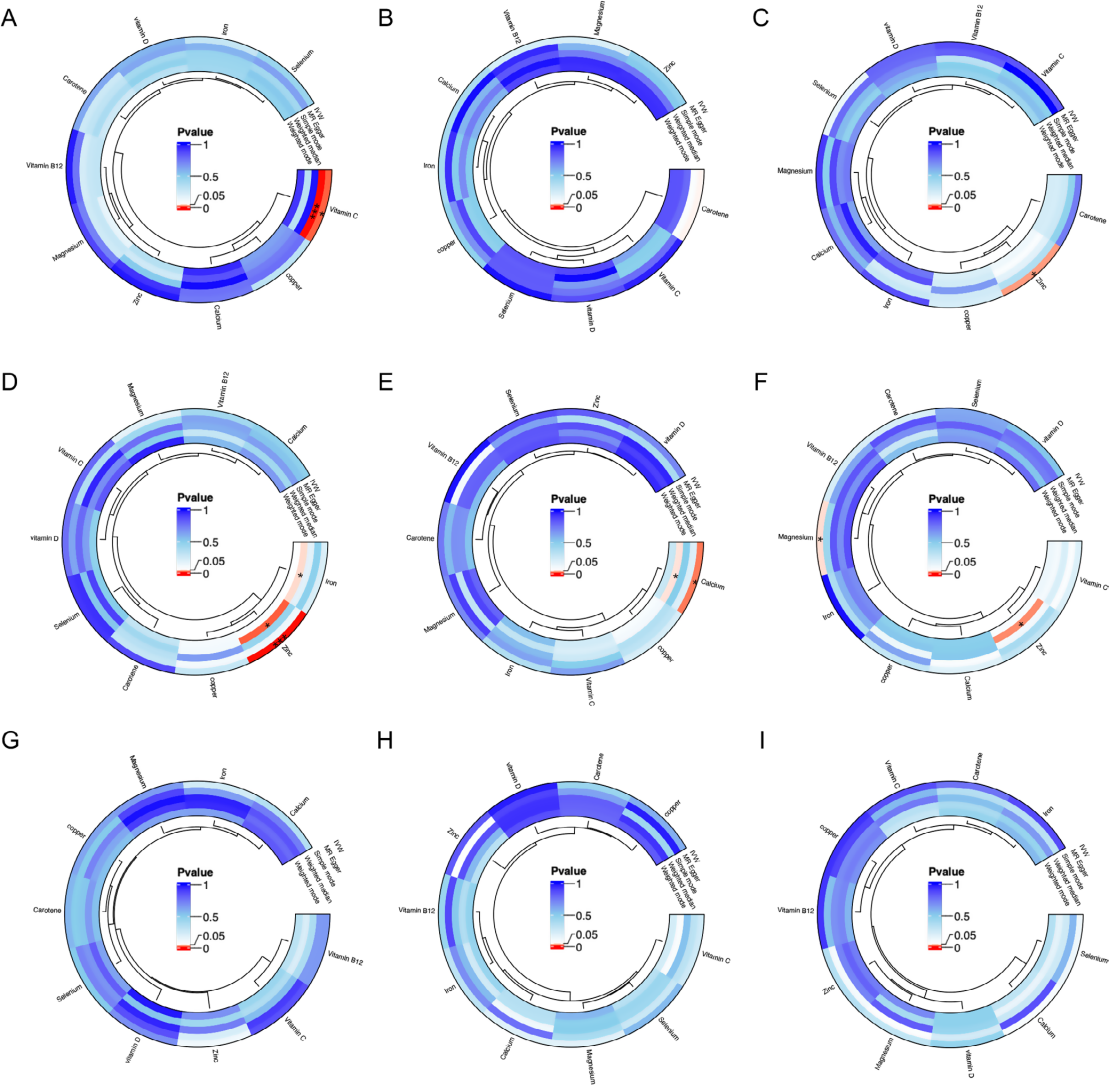
Causal effect of circulating micronutrients (calcium, iron, zinc, copper, magnesium, selenium, carotene, vitamin B12, vitamin C, and vitamin D) on ulcer risks using a bidirectional two‐sample Mendelian randomization (MR) approach. (A) Corneal ulcer, (B) recurring oral aphthae, (C) esophageal ulcer, (D) gastric ulcer, (E) duodenal ulcer, (F) vaginal/vulvar ulcer, (G) decubitus ulcer, (H) lower limb ulcer, and (I) chronic skin ulcer. Statistically significant associations are indicated with asterisks: *p < 0.05, ***p < 0.001. The corrected threshold for statistical significance for multiple tests was set at *p* = 0.05/10 = 0.005. The *p* value of 0.005 to 0.05 is considered nominally significant.

**FIGURE 3:**
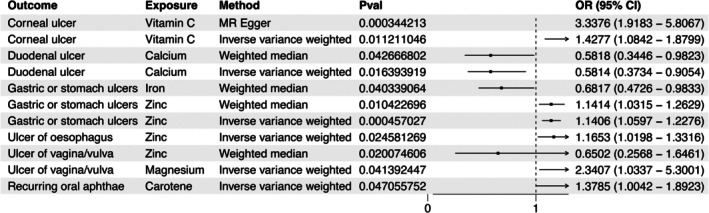
Forest plot for the causal effect of circulating micronutrients (calcium, iron, zinc, copper, magnesium, selenium, carotene, vitamin B12, vitamin C, and vitamin D) on the risks of various ulcers (corneal ulcer, recurring oral aphthae, esophageal ulcer, gastric ulcer, duodenal ulcer, vaginal/vulvar ulcer, decubitus ulcer, lower limb ulcer, and chronic skin ulcer). The corrected threshold for statistical significance for multiple tests was set at *p* = 0.05/10 = 0.005. The *p* value of 0.005 to 0.05 is considered nominally significant.

**FIGURE 4:**
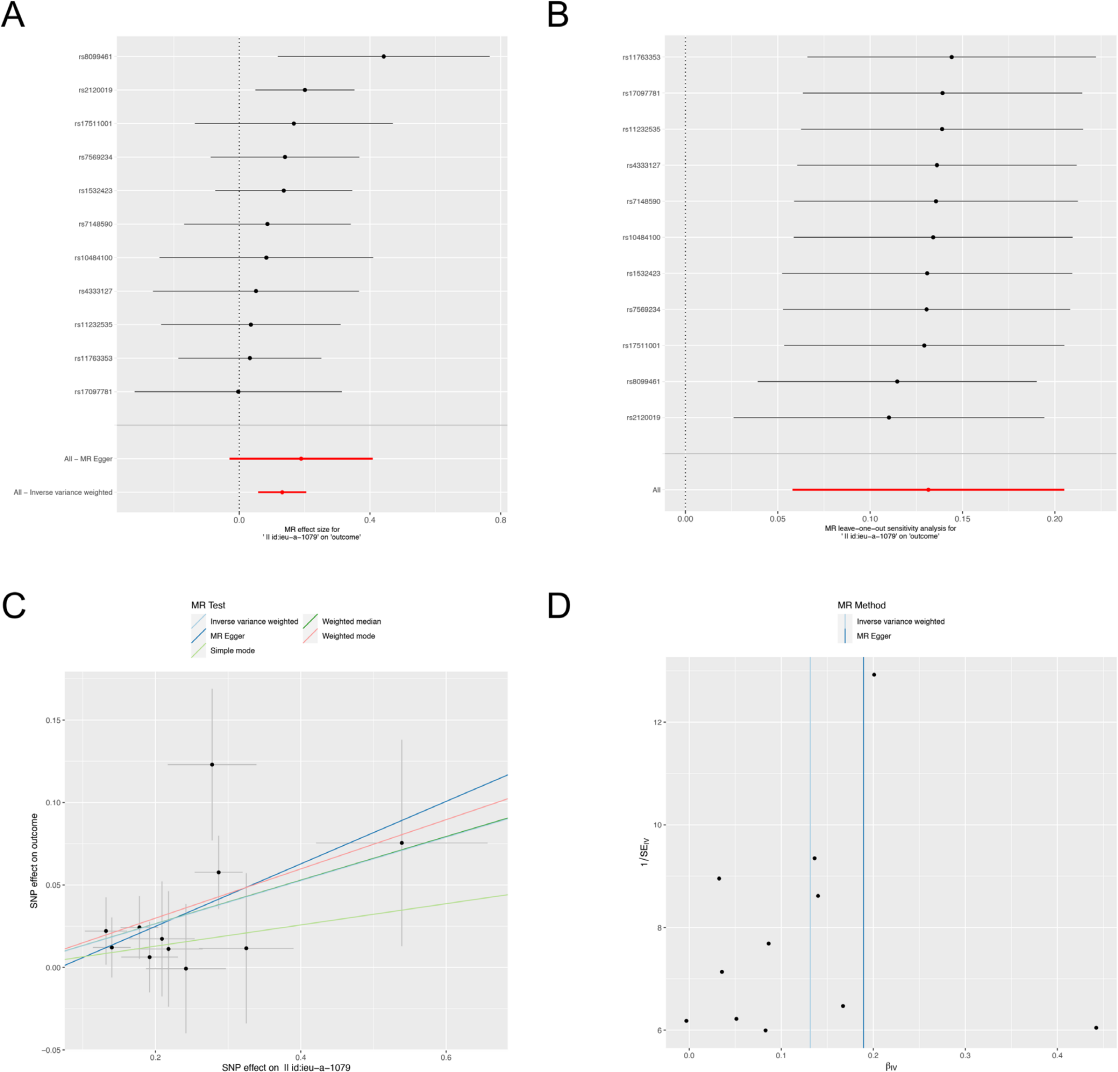
Mendelian randomization (MR) for the causal effect of circulating micronutrient concentrations on gastric ulcer risk. (A) Forest plot, (B) leave‐one‐out sensitivity analysis, (C) scatter plot, and (D) funnel plot of the genetic risk of circulating zinc on gastric ulcer.

The influence of circulating micronutrient concentrations on ulcer risk was evaluated using the IVW, MR‐Egger, WM, simple mode, and weighted mode methods, depicted in Figure 2. After correcting for multiple testing, the only statistically significant association between micronutrients and ulcers was observed for genetically predicted blood zinc concentration and gastric ulcer risk (Figure 3 and Table 1). A positive association was observed, where a standard deviation increase in circulating zinc concentration was associated with an OR of 1.141 (IVW, 95% CI: 1.060–1.228, *p* = 4.57E‐4) (Figures 3 and 4A and Table 1). A nominally significant association was also found between genetically predicted circulating zinc concentration and esophageal ulcer risk, with an OR of 1.165 (IVW, 95% CI: 1.020–1.332, *p* = 0.025) (Figure 3 and Table 1). In addition, a nominally significant association was observed for genetically predicted vitamin C concentration and corneal ulcer risk with an OR of 1.428 (IVW, 95% CI: 1.084–1.880, *p* = 0.011) (Figure 3). Genetically predicted carotene concentration was observed to be associated with the risk of recurring oral aphthae with an OR of 1.378 (IVW, 95% CI: 1.004–1.892, *p* = 0.047) (Figure 3 and Table 1). Circulating magnesium concentration was linked to the risk of vaginal/vulvar ulcers with an OR of 2.341 (IVW, 95% CI: 1.034–5.300, *p* = 0.041) (Figure 3 and Table 1). Nevertheless, these findings are considered nominal and exploratory due to their lack of significance after multiple testing corrections. Additionally, a suggestively negative association was observed for calcium concentration and duodenal ulcer risk with an OR of 0.581 (IVW, 95% CI: 0.373–0.905, *p* = 0.016) (Figure 3 and Table 1). At the same time, a negative association was found for circulating iron concentration and gastric ulcer risk with an OR of 0.682 (WM, 95% CI: 0.473–0.983, *p* = 0.040) (Figure 3). A nominal negative correlation was also found between circulating zinc concentration and vaginal/vulvar ulcer risk with an OR of 0.674 (WM, 95% CI: 0.484–0.940, *p* = 0.020) (Figure 3). No correlation was identified between ulcer risk and other micronutrients. The 10 micronutrients examined in this research demonstrated no statistical association with decubitus ulcers, lower limb ulcers, or chronic skin ulcers.

**TABLE 1:** Mendelian randomization (MR) analyses of circulating micronutrients on the risk of ulcers.

Micronutrients	Ulcers	Odds ratio (95% CI)	*p*
Zinc	Gastric ulcer	1.141 (1.060–1.228)	4.57E‐04
Zinc	Esophagus ulcer	1.165 (1.020–1.332)	0.025
Zinc	Vagina/vulva ulcer	0.674 (0.484–0.940)	0.020
Carotene	Recurring oral aphthae	1.378 (1.004–1.892)	0.047
Iron	Gastric ulcer	0.682 (0.473–0.983)	0.040
Calcium	Duodenal ulcer	0.581 (0.373–0.905)	0.016
Magnesium	Vagina/vulva ulcer	2.341 (1.034–5.300)	0.041

### 4.2 MR Sensitivity Evaluation

Detailed information on sensitivity analysis can be found in Table S3. The Cochrane's Q‐test results indicate that there may be heterogeneity in some IVs. When studying the relationship between iron and gastric ulcers, heterogeneity among IVs may exist (*P*_Cochran's Q_ = 0.043). Similarly, when examining the association between copper and gastric ulcers, heterogeneity among IVs is also present (*P*_Cochran's Q_ = 0.006). Similarly, when investigating the relationship between vitamin B12 and gastric ulcers, similar situations may occur (*P*_Cochran's Q_ = 0.009). Additionally, potential heterogeneity among IVs is detected when exploring the connections between copper and duodenal ulcers (*P*_Cochran's Q_ = 0.021), vitamin B12 and duodenal ulcers (*P*_Cochran's Q_ = 0.016), magnesium and duodenal ulcers (*P*_Cochran's Q_ = 0.036), calcium and duodenal ulcers (*P*_Cochran's Q_ = 0.031), as well as magnesium and vulvar ulcers (*P*_Cochran's Q_ = 0.042). No heterogeneity among IVs was detected for other variables used in the respective studies.

Our analysis revealed a significant and positive association between genetically predicted zinc concentration and gastric ulcer risk (Figure 4A). The leave‐one‐out analysis showed no outliers (Figure 4B). The scatterplot revealed the consistent direction of the estimated MR effect for IVW, MR Egger, weighted mode, simple mode, and WM method (Figure 4C). Based on MR‐Egger regression analysis, we found some evidence for horizontal pleiotropy in the analyses (Table S3). IVs may exhibit pleiotropy in the relationship between vitamin C and corneal ulcer (*P*_MR‐Egger intercept_ = 0.002), zinc and lower limb ulcer (*P*_MR‐Egger intercept_ = 0.044), and zinc and chronic skin ulcer (*P*_MR‐Egger intercept_ = 0.048). The funnel plots showed no significant heterogeneity for both IVW and MR Egger models in the analysis of zinc concentration and gastric ulcer risk (Figure 4D).

### 4.3 CAUSE Analysis as Complementary Validation

To further evaluate the robustness of selected positive MR associations, we applied MR‐CAUSE analysis to five exposure–outcome pairs that demonstrated suggestive evidence of causality in two‐sample MR, including zinc–stomach ulcer (Figure S1), zinc–esophageal ulcer (Figure S2), magnesium–vaginal/vulvar ulcer (Figure S3), calcium–duodenal ulcer (Figure S4), and vitamin C–corneal ulcer (Figure S5).

Although the causal model generally demonstrated better fit compared to null or sharing models, the posterior distributions of the causal effect parameter (gamma) were consistently centered around zero with wide confidence intervals. For example, the estimated gamma for zinc–stomach ulcer was 0 (95% CI: −0.1 to 0.1), and for zinc–esophageal ulcer was −0.01 (95% CI: −0.19 to 0.17), suggesting limited support for a strong causal relationship under the CAUSE framework. These findings indicate that while traditional MR methods suggested potential associations, CAUSE—a more conservative, pleiotropy‐aware model—did not provide strong statistical evidence for causality in these specific micronutrient–ulcer pairs.

### 4.4 MR‐Clust Analysis

MR‐Clust was applied to all micronutrient–ulcer pairs with nominally significant MR associations (Figure S6). Among these, only the vitamin C–corneal ulcer pair showed evidence of clustering, with SNPs separated into three distinct clusters. One cluster (Cluster 1) exhibited a negative slope suggestive of a protective effect, while another (Cluster 2) showed a clear positive slope. The third cluster (Cluster 3) included variants with weak or null associations. These results suggested the presence of heterogeneous effects among SNPs used as instruments for circulating vitamin C levels, potentially reflecting different biological pathways or pleiotropic effects influencing corneal ulcer risk. No distinct clusters were observed for other element–ulcer pairs, indicating a lack of heterogeneity in those associations under the MR‐Clust model.

## 5 Discussion

In this study, we utilized summary statistics from genetic research and large consortia to investigate the causal relationships between ten circulating micronutrient concentrations and the occurrence of nine distinct types of ulcers. We identified a significant association between genetically predicted circulating zinc levels and the risk of gastric ulcers. Additionally, we observed nominal associations between other micronutrients and various ulcers. To enhance the robustness and interpretability of our findings, we incorporated pleiotropy‐aware modeling and SNP clustering analyses. Although the CAUSE framework did not confirm strong causality for most pairs, MR‐Clust revealed three distinct SNP clusters in the association between vitamin C and corneal ulcers, suggesting potential mechanistic heterogeneity. Overall, this study provides a comprehensive overview of the relationships between trace elements and ulcers, emphasizing zinc as a critical candidate for further mechanistic investigation in gastric ulcer pathogenesis.

Zinc is an essential trace element vital for numerous biological functions, including DNA and protein synthesis (Saper and Rash 2009), and has been studied for its therapeutic potential in gastric ulcers. While previous research highlighted zinc's role in healing gastric ulcers, Mei et al. found that Zn(II)‐curcumin accelerated the healing of acetic acid‐induced chronic gastric ulcers in rats by reducing oxidative stress and downregulating matrix metalloproteinase 9 (MMP9) (Mei et al. 2013). Polaprezinc, a chelating compound composed of zinc and L‐carnosine, has been found to promote the healing of gastric ulcers by enhancing the repair and protection of the gastric mucosa, reducing gastric acid secretion, and increasing mucin and bicarbonate secretion (Efthymakis and Neri 2022; Hewlings and Kalman 2020). In addition, Yu et al. discovered that zinc taurine could protect rat gastric mucosa from cold stress‐induced ulcers by reducing oxidative stress, promoting endogenous expression of HSP70, and alleviating psychological stress (Yu et al. 2015).

Our MR study indicates that increased circulating zinc concentration may be a risk factor for gastric ulcers. This contrasts with earlier studies focused on therapeutic effects post‐ulcer development, which did not directly assess the impact of serum zinc levels on ulcer incidence. For instance, a retrospective study at Okayama University Hospital revealed a 31.0% ulcer occurrence rate among patients taking zinc acetate dihydrate, suggesting a possible link between increased serum zinc and ulcer formation (Iwamuro et al. 2022). Additionally, case studies and analyses in patients with Wilson's disease further support the notion that prolonged zinc supplementation may contribute to gastric ulcers (Gilbert et al. 2016). Notably, a large‐scale study found a positive correlation between zinc in rainwater and hospitalization rates for gastric ulcers (Tubek et al. 2011), aligning with our MR findings. These findings suggest a complex dual role of zinc in gastric health. On one hand, zinc can protect mucosal integrity and modulate inflammation (Yu et al. 2025); on the other hand, excessive zinc levels—depending on dosage, tissue context, and pathological state—may lead to adverse effects such as oxidative stress and metal toxicity, which can facilitate infections like those caused by *Helicobacter pylori* (Rihacek et al. 2023; Wu et al. 2021). This complexity underscores the need for careful consideration of zinc supplementation, particularly in populations at risk for gastric mucosal injury. Future research integrating molecular, clinical, and epidemiological approaches is warranted to elucidate the mechanisms governing zinc's dual effects in gastric ulcer pathogenesis. Clinicians should remain vigilant regarding the potential hazards of elevated zinc exposure and implement appropriate preventive strategies when recommending zinc supplementation.

Currently, there is no research establishing a relationship between circulating zinc concentration and vaginal or vulvar ulcers. We are the first to report a nominal association between increased zinc concentration and vaginal ulcers, highlighting the need for further experimental research to validate this finding. One study indicated that zinc acetate, when used as a vaginal contraceptive, does not cause epithelial ulcers in the vagina (Fahim and Wang 1996). This suggests that zinc may elicit different responses in gastric and vaginal epithelial tissues. In our study, we observed opposing trends in zinc's effects on gastric ulcers compared to vaginal ulcers, which may reflect variations in zinc metabolism and mechanisms of action across different tissues. A plausible explanation lies in the tissue‐specific biology of zinc: in the gastrointestinal mucosa, excess zinc can alter gastric acid secretion, disrupt epithelial tight junctions, and modify the local microbiota, thereby impairing mucosal defense (Wu et al. 2021). Conversely, in vaginal and other skin‐like epithelia, zinc's anti‐inflammatory, antimicrobial, and wound‐healing properties may predominate, enhancing epithelial barrier integrity and providing protection against ulceration (Lin et al. 2017). These divergent effects may also be influenced by differences in epithelial structure, local immune environments, and zinc transporter expression among tissues. Given the complexity of zinc metabolism and regulation, further mechanistic studies are essential to clarify the pathways underlying these site‐specific associations.

Our results indicate that increased calcium may serve as a nominal protective factor against duodenal ulcers. Calcium is a common electrolyte in the mucous layer secreted by duodenal epithelial cells, and the release of mucins requires calcium‐mediated exocytosis (Galura et al. 2019). This mucous layer serves to protect the underlying tissue from ulcer damage, suggesting that elevated circulating calcium may indeed confer protection against duodenal ulceration. Additionally, we have identified, for the first time, a nominal association between elevated circulating carotene levels and the risk of recurrent oral aphthae. Carotene is a potent antioxidant that typically protects skin and mucous membranes from oxidative stress damage (Ma et al. 2025). However, while carotene possesses antioxidant properties, excessive levels of antioxidants can lead to adverse effects, disrupting the balance of oxidative stress in the body (Sarangarajan et al. 2017). This imbalance may harm the oral mucosa and increase the risk of ulceration. Furthermore, carotene is involved in regulating immune function (Arshad et al. 2025). We hypothesize that high levels of carotene may disrupt immune responses, affecting normal cellular metabolism and regeneration, potentially triggering ulcer formation. In future research, we will focus on the immune system to thoroughly investigate the mechanisms by which carotene levels influence the occurrence of recurrent oral aphthae.

Chronic skin ulcers, lower limb ulcers, and pressure ulcers are persistent lesions affecting skin tissue, often challenging to heal and prone to recurrence. Individuals who are bedridden, engage in prolonged standing, or have limited mobility, as well as those with chronic conditions like diabetes or vascular diseases, are at higher risk for these ulcers. Treatment typically requires long‐term management, incorporating holistic approaches such as wound care, systemic therapies, and nutritional support. Micronutrient imbalances are common among patients with chronic ulcers, leading to extensive debate regarding the role of circulating micronutrients in their treatment. A systematic review and meta‐analysis indicated benefits from supplementation with vitamin C, copper, selenium, and zinc for pressure ulcers (Saeg et al. 2021). Diabetic ulcers have shown improvements with vitamins A, D, E, and magnesium, while venous ulcers appear responsive to zinc supplementation. However, RCTs have contested these findings, with some showing that vitamin C supplementation does not accelerate pressure ulcer healing (ter Riet et al. 1995) and that nutritional interventions may not prevent pressure ulcers (Houwing et al. 2003). Another study also indicates that there is currently no clear evidence to suggest that nutritional interventions can reduce the development of pressure ulcers or aid in their healing (Langer and Fink 2014). Despite this, it is essential to recognize that the quality of existing evidence is generally low. Recent insights suggest that adding micronutrient supplements to the diet may not significantly differ from a regular diet in preventing pressure ulcers (Langer et al. 2024). Additionally, a meta‐analysis found that reduced levels of vitamins C, D, magnesium, and selenium increased the risk of diabetic foot ulcers, while changes in calcium, iron, copper, zinc, and vitamin B12 did not appear to affect this risk (Kurian et al. 2023). Our MR study supports these findings by revealing no causal effect of genetically predicted circulating levels of vitamins C, D, magnesium, and selenium on chronic skin and lower limb ulcers, suggesting that supplementation with certain micronutrients may have limited efficacy in preventing specific skin ulcers. Given the complex and multifactorial nature of ulcer pathology, further research is needed to elucidate the role of circulating micronutrients in ulcer development and healing.

To further validate the robustness of our MR findings, we employed two advanced sensitivity methods—CAUSE and MR‐Clust—to assess potential pleiotropy and heterogeneity among the instruments. The CAUSE model, being conservative and accounting for both correlated and uncorrelated pleiotropy, did not identify strong causal signals for the five selected element‐ulcer pairs that had shown nominal significance in traditional MR. The posterior estimates of the causal effect were centered around zero with wide credible intervals, indicating limited evidence of robust causality under the CAUSE framework. In contrast, MR‐Clust was used to investigate SNP‐level heterogeneity. Notably, only the vitamin C–corneal ulcer pair exhibited distinct SNP clustering, revealing three separable clusters with heterogeneous causal signals. One cluster demonstrated a strong positive effect, while another suggested a potential inverse effect. This finding supports the existence of heterogeneous mechanisms for vitamin C and corneal ulcers, despite the presence of pleiotropy.

Literature indicates that vitamin C is crucial for collagen synthesis, essential for wound healing and tissue regeneration (Palmieri et al. 2019). As a key component of connective tissue, collagen maintains the structure and function of the cornea. Additionally, vitamin C exhibits immune‐modulating properties that enhance macrophage function and regulate inflammatory responses, further influencing corneal wound healing (Kwok et al. 2019; Li et al. 2021). The SNP clustering observed for the vitamin C–corneal ulcer pair suggests that different genetic variants may affect corneal ulcer risk through distinct biological pathways. While similar clustering was not observed in other exposure–outcome pairs, these findings underscore the complexity of genetic architecture and the need for cautious interpretation. By integrating traditional MR with modern sensitivity analyses, we gain a more comprehensive understanding of the role of circulating micronutrients in ulcer development and open new avenues for future research.

Our studies have several advantages. First, we systematically explored the correlation between circulating micronutrients and ulcer risk through comprehensive MR analysis. This study represents the first large‐scale genetic investigation to reveal a causal relationship between circulating micronutrients and ulcers. Second, our MR approach uses genetic variables as a research tool to mitigate biases inherent in observational studies. We assessed participants of European ancestry to reduce the risk of population stratification. By combining summary data from multiple cohorts, we significantly reduced random errors and increased statistical power. We also used the PhenoScanner database to eliminate confounding factors, ensuring the independent assumption of IVs. Third, this MR study has a large sample size and ensures robust estimation effects for each instrumental variable, with F statistics > 10, effectively safeguarding the statistical power of this study. Finally, our findings were supported by the execution of various sensitivity analyses. Cochrane's Q and MR‐Egger intercept test were employed to further assess and mitigate pleiotropy.

The current study has several notable limitations that must be acknowledged. First, the selection of IVs was based on a *p* value threshold of ≤ 1e‐5. While this threshold allows for a broader range of SNPs to be included in the analysis, it may also introduce false‐positive IVs that could compromise the validity of the findings. To enhance rigor, we recognize the importance of conducting sensitivity analyses with stricter thresholds (e.g., *p* < 5e‐8). However, adopting such stringent criteria may significantly reduce the number of available SNPs for analysis, potentially limiting the power to detect true associations. We plan to validate our findings in future research using renewed and larger datasets, which will allow for a more robust assessment of these associations. Second, the study's scope was restricted to individuals of European ancestry, which may limit the generalizability of our findings to other ethnic groups. This restriction raises concerns about potential ancestry‐specific effects that could influence the outcomes. To enhance the credibility and applicability of our results, future research should aim to include diverse populations, allowing for a more comprehensive understanding of the associations across different genetic backgrounds. Third, the reliance on summary data precluded stratification by factors such as sex, age, diet, and nutritional supplement use. This limitation may have led to overlapping information among the populations studied, including those with decubitus ulcers, lower limb ulcers, and chronic skin ulcers. Additionally, the inability to identify individuals with multiple ulcer types may have introduced bias into our findings. Furthermore, we acknowledge the risk of weak instrument bias associated with the zinc GWAS, which included only 2603 participants compared to approximately 65,000 for other micronutrients. Although F‐statistics > 10 were reported, we recognize the potential for weak instruments and the need for validation using larger zinc GWAS studies, such as those from the UK Biobank, in our future research. Finally, while we employed various methods to mitigate the influence of pleiotropy, the potential impact of unaccounted pleiotropic effects on our results cannot be entirely excluded.

## 6 Conclusions

In conclusion, this study established a genetic correlation between circulating micronutrients and ulcer development, focusing specifically on the impact of circulating zinc on gastric ulcers. Further correlations were identified, such as the association between circulating zinc on esophageal and vaginal/vulvar ulcers, iron on gastric ulcers, magnesium on vaginal/vulvar ulcers, carotene on recurrent oral aphthae, and calcium on duodenal ulcers. While CAUSE did not confirm robust causality in most pairs, MR‐Clust uncovered three distinct SNP clusters in the vitamin C‐corneal ulcer pair, indicating potential mechanistic heterogeneity. Collectively, this study offers a panoramic view of trace element–ulcer relationships and prioritizes zinc as a key candidate for further mechanistic exploration in gastric ulcer pathogenesis.

## Author Contributions

**Xueyao Cai:** conceptualization (equal), methodology (equal), writing – original draft (equal). **Weidong Li:** conceptualization (equal), investigation (equal), resources (equal). **Wenjun Shi:** investigation (equal), writing – review and editing (equal). **Can Liu:** methodology (equal), supervision (equal). **Yuchen Cai:** methodology (equal), supervision (equal), writing – review and editing (equal). **Jianda Zhou:** project administration (lead), supervision (equal), writing – review and editing (equal).

## Conflicts of Interest

The authors declare no conflicts of interest.

## Supporting information


**Figure S1:** Mendelian randomization (MR) CAUSE analysis for causal estimates of circulating zinc on stomach ulcer risk. The parameter gamma represents the causal effect estimate.
**Figure S2:** Mendelian randomization (MR) CAUSE analysis for causal estimates of circulating zinc on esophageal ulcer risk. The parameter gamma represents the causal effect estimate.
**Figure S3:** Mendelian randomization (MR) CAUSE analysis for causal estimates of circulating magnesium on vaginal/vulvar ulcer risk. The parameter gamma represents the causal effect estimate.
**Figure S4:** Mendelian randomization (MR) CAUSE analysis for causal estimates of circulating calcium on duodenal ulcer risk. The parameter gamma represents the causal effect estimate.
**Figure S5:** Mendelian randomization (MR) CAUSE analysis for causal estimates of circulating vitamin C on corneal ulcer risk. The parameter gamma represents the causal effect estimate.
**Figure S6:** Mendelian randomization (MR) Clust analysis for causal estimates of vitamin C on corneal ulcer. (A) Cluster 1 showed a negative slope suggestive of a protective effect. (B) Cluster 2 showed a positive slope suggestive of a risk effect. (C) Cluster 3 included variants that show weak or null associations with corneal ulcer risk.

**Table S1:** Supporting information for 224 SNPs used as genetic instruments in Mendelian randomization analyses of 10 circulating micronutrients. Table S2. The Mendelian randomization analyses of 10 circulating micronutrients and the risk of nine ulcers. Table S3. Cochran's Q test and MR‐Egger intercept of 10 circulating micronutrients and the risk of nine ulcers.

**File S1:** fsn371094‐sup‐0003‐FileS1.docx.

## Data Availability

All data can be requested from the corresponding authors. The R code used for MR‐CAUSE and MR‐Clust is available on GitHub (https://github.com/kevinkevinkevin666/code) and in the Supporting Information.
