# Supplementary material for: A Trace Element–Ulcer Map: Decoding Micronutrient–Ulcer Relationships Through Genetic Architecture and Pleiotropy‐Aware Inference
Source: Food Sci Nutr. 2025 Oct 31;13(11):e71094. doi: 10.1002/fsn3.71094 (PMC12576808; doi:10.1002/fsn3.71094)
Supplement: Supplementary file 1 — Figure S1: Mendelian randomization (MR) CAUSE analysis for causal estimates of circulating zinc on stomach ulcer risk. The parameter gamma represents the causal effect estimate. Figure S2: Mendelian randomization (MR) CAUSE analysis for causal estimates of circulating zinc on esophageal ulcer risk. The parameter gamma represents the causal effect estimate. Figure S3: Mendelian randomization (MR) CAUSE analysis for causal estimates of circulating magnesium on vaginal/vulvar ulcer risk. The parameter gamma represents the causal effect estimate. Figure S4: Mendelian randomization (MR) CAUSE analysis for causal estimates of circulating calcium on duodenal ulcer risk. The parameter gamma represents the causal effect estimate. Figure S5: Mendelian randomization (MR) CAUSE analysis for causal estimates of circulating vitamin C on corneal ulcer risk. The parameter gamma represents the causal effect estimate. Figure S6: Mendelian randomization (MR) Clust analysis for causal estimates of vitamin C on corneal ulcer. (A) Cluster 1 showed a negative slope suggestive of a protective effect. (B) Cluster 2 showed a positive slope suggestive of a risk effect. (C) Cluster 3 included variants that show weak or null associations with corneal ulcer risk. [file FSN3-13-e71094-s003.docx]

**Supplementary Figures**

**Figure S1.** Mendelian randomization (MR) CAUSE analysis for causal estimates of circulating zinc on stomach ulcer risk. The parameter gamma represents the causal effect estimate.

**Figure S2.** Mendelian randomization (MR) CAUSE analysis for causal estimates of circulating zinc on esophageal ulcer risk. The parameter gamma represents the causal effect estimate.

**Figure S3.** Mendelian randomization (MR) CAUSE analysis for causal estimates of circulating magnesium on vaginal/vulvar ulcer risk. The parameter gamma represents the causal effect estimate.

**Figure S4.** Mendelian randomization (MR) CAUSE analysis for causal estimates of circulating calcium on duodenal ulcer risk. The parameter gamma represents the causal effect estimate.

**Figure S5.** Mendelian randomization (MR) CAUSE analysis for causal estimates of circulating vitamin C on corneal ulcer risk. The parameter gamma represents the causal effect estimate.


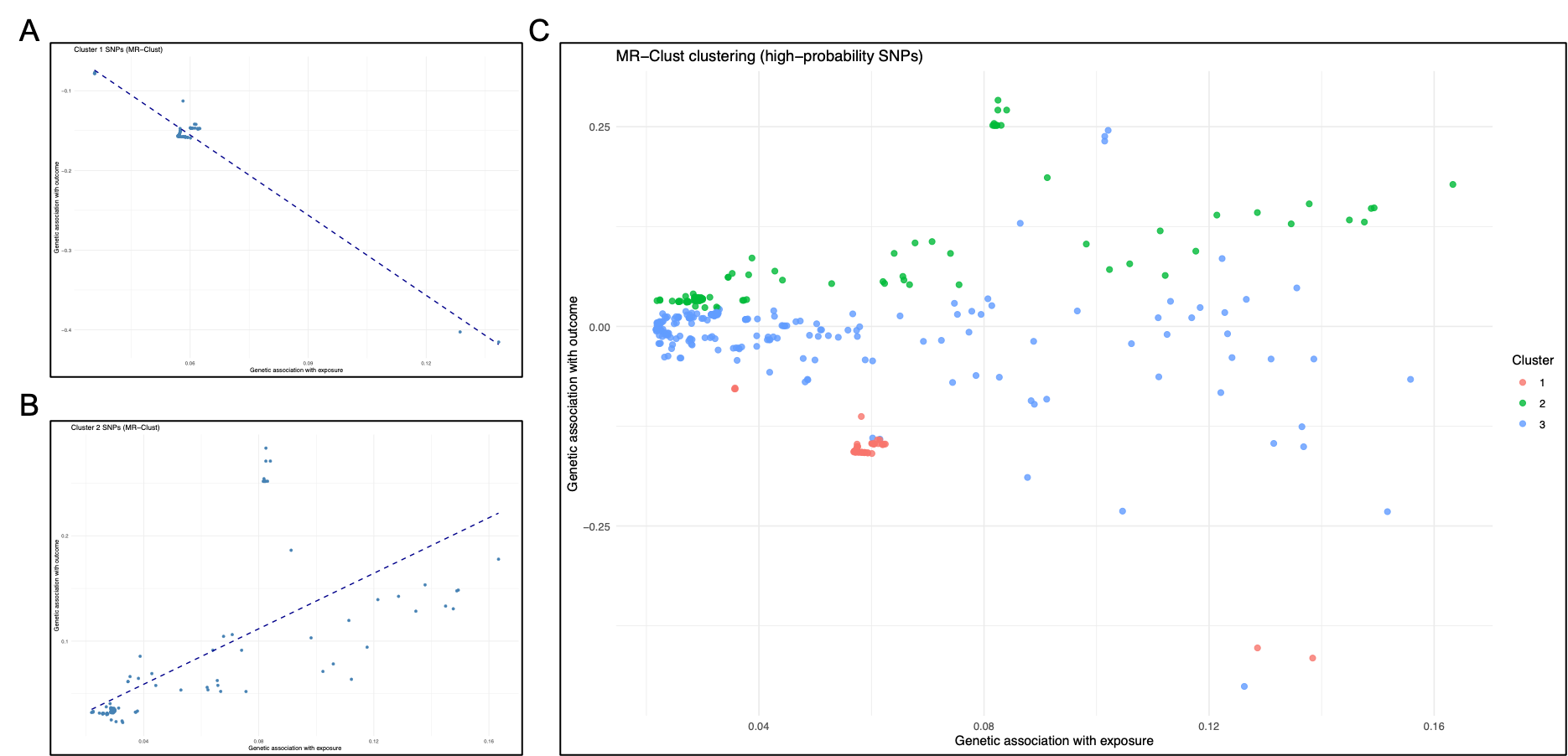


**Figure S6.** Mendelian randomization (MR) Clust analysis for causal estimates of vitamin C on corneal ulcer. (A) Cluster 1 showed a negative slope suggestive of a protective effect. (B) Cluster 2 showed a positive slope suggestive of a risk effect. (C) Cluster 3 included variants that show weak or null associations with corneal ulcer risk.
