# Supplementary material for: A Trace Element–Ulcer Map: Decoding Micronutrient–Ulcer Relationships Through Genetic Architecture and Pleiotropy‐Aware Inference
Source: Food Sci Nutr. 2025 Oct 31;13(11):e71094. doi: 10.1002/fsn3.71094 (PMC12576808; doi:10.1002/fsn3.71094)
Supplement: Supplementary file 3 — File S1: fsn371094‐sup‐0003‐FileS1.docx. [file FSN3-13-e71094-s002.docx]

**MR-CAUSE**

library(cause)

library(data.table)

library(dplyr)

library(ieugwasr)

library(genetics.binaRies)

# === Step 1===

gwas1 <- fread("a.txt.gz", header = TRUE)

gwas2 <- fread("b.gz", header = TRUE)

# === Step 2===

dat <- gwas_merge(

gwas1, gwas2,

snp_name_cols = c("SNP", "rsids"),

beta_hat_cols = c("b", "beta"),

se_cols = c("se", "sebeta"),

A1_cols = c("A1", "alt"),

A2_cols = c("A2", "ref")

)

# === Step 3===

variants <- dat %>%

mutate(pval1 = 2 * pnorm(abs(beta_hat_1 / seb1), lower.tail = FALSE))

# === Step 4===

ld_dir <- ""

all_top_vars <- c()

for (chr in 1:22) {

ld_path <- file.path(ld_dir, sprintf("chr%d_LD.RDS", chr))

info_path <- file.path(ld_dir, sprintf("chr%d_info.RDS", chr))

if (!file.exists(ld_path) || !file.exists(info_path)) {

message(sprintf("❌ chr%d skipped (LD or info file not found)", chr))

next

}

ld <- readRDS(ld_path)

snp_info <- readRDS(info_path)

chr_variants <- variants[variants$snp %in% snp_info$SNP, ]

if (nrow(chr_variants) < 100) next

pruned <- tryCatch({

cause::ld_prune(

variants = chr_variants,

ld = ld,

total_ld_variants = snp_info$SNP,

pval_cols = "pval1",

pval_thresh = 0.05,

r2_thresh = 0.05

)

}, error = function(e) NA)

if (!is.null(pruned) && !all(is.na(pruned))) {

all_top_vars <- c(all_top_vars, pruned)

}

}

top_vars <- unique(all_top_vars)

cat("✅ SNP:", length(top_vars), "\n")

# === Step 5==

set.seed(100)

varlist <- sample(dat$snp, size = min(200000, nrow(dat)), replace = FALSE)

# === Step 6===

res <- cause(X = dat, variants = top_vars, param_ests = params, force = TRUE)

# === Step 7: 输出结果 ===

summary(res)

plot(res)

library(cause)

library(data.table)

library(dplyr)

library(ieugwasr)

library(genetics.binaRies)

# === Step 1===

gwas1 <- fread("a.txt.gz", header = TRUE)

gwas2 <- fread("b.gz", header = TRUE)

# === Step 2 ===

dat <- gwas_merge(

gwas1, gwas2,

snp_name_cols = c("SNP", "rsids"),

beta_hat_cols = c("b", "beta"),

se_cols = c("se", "sebeta"),

A1_cols = c("A1", "alt"),

A2_cols = c("A2", "ref"),

)

# === Step 3 ===

variants <- dat %>%

mutate(pval1 = 2 * pnorm(abs(beta_hat_1 / seb1), lower.tail = FALSE))

# === Step 4 ===

ld_dir <- ""

all_top_vars <- c()

for (chr in 1:22) {

ld_path <- file.path(ld_dir, sprintf("chr%d_LD.RDS", chr))

info_path <- file.path(ld_dir, sprintf("chr%d_info.RDS", chr))

if (!file.exists(ld_path) || !file.exists(info_path)) {

message(sprintf("❌ chr%d skipped (LD or info file not found)", chr))

next

}

ld <- readRDS(ld_path)

snp_info <- readRDS(info_path)

chr_variants <- variants[variants$snp %in% snp_info$SNP, ]

if (nrow(chr_variants) < 100) next

pruned <- tryCatch({

cause::ld_prune(

variants = chr_variants,

ld = ld,

pval_cols = "pval1",

pval_thresh = 1e-3,

r2_thresh = 0.01

)

}, error = function(e) NA)

if (!is.null(pruned) && !all(is.na(pruned))) {

all_top_vars <- c(all_top_vars, pruned)

}

}

top_vars <- unique(all_top_vars)

cat("✅ 全基因组剪枝后 SNP 数量:", length(top_vars), "\n")

# === Step 5===

set.seed(100)

varlist <- sample(dat$snp, size = min(200000, nrow(dat)), replace = FALSE)

# === Step 6 ===

res <- cause(X = dat, variants = top_vars, param_ests = params, force = TRUE)

# === Step 7===

summary(res)

plot(res)

**MR-Clust**

library(mrclust)

library(data.table)

library(ggplot2)

library(TwoSampleMR)

# Step 1

exposure_dat <- read_exposure_data("a.txt.gz",

sep = "\t",

snp_col = "SNP",

beta_col = "b",

se_col = "se",

effect_allele_col = "A1",

other_allele_col = "A2",

eaf_col = "p",

pval_col = "p")

outcome_dat <- read_outcome_data("b.gz",

snps = exposure_dat$SNP,

sep = "\t",

snp_col = "rsids",

beta_col = "beta",

se_col = "sebeta",

effect_allele_col = "alt",

other_allele_col = "ref",

eaf_col = "af_alt",

pval_col = "pval")

# Step 2: harmonise

harmonised <- harmonise_data(exposure_dat, outcome_dat)

# === Step 3===

library(data.table)

setDT(harmonised)

harmonised <- harmonised[

!is.na(beta.exposure) &

!is.na(beta.outcome) &

!is.na(se.exposure) &

!is.na(se.outcome)

]

# === Step 4 ===

bx <- harmonised$beta.exposure

bxse <- harmonised$se.exposure

by <- harmonised$beta.outcome

byse <- harmonised$se.outcome

theta <- by / bx

theta_se <- byse / abs(bx)

# === Step 5===

library(mrclust)

res_em <- mr_clust_em(

theta = theta,

theta_se = theta_se,

bx = bx,

by = by,

bxse = bxse,

byse = byse,

obs_names = snp_names

)

# === Step 6===

head(res_em$results$best)

library(ggplot2)

res_plot <- res_em$results$best

res_plot$bx <- harmonised$beta.exposure[match(res_plot$observation, harmonised$SNP)]

res_plot$by <- harmonised$beta.outcome[match(res_plot$observation, harmonised$SNP)]

ggplot(res_plot, aes(x = bx, y = by, color = as.factor(cluster))) +

geom_point(size = 2, alpha = 0.8) +

theme_minimal() +

labs(

x = "Genetic association with exposure",

y = "Genetic association with outcome",

color = "Cluster"

) +

ggtitle("MR-Clust clustering (manual plot)")
